# Comparative genomics reveals a lack of evidence for pigeons as a main source of *stx*_2f_*-*carrying *Escherichia coli* causing disease in humans and the common existence of hybrid Shiga toxin-producing and enteropathogenic *E. coli* pathotypes

**DOI:** 10.1186/s12864-019-5635-z

**Published:** 2019-04-05

**Authors:** Angela H. A. M. van Hoek, Janieke N. J. van Veldhuizen, Ingrid Friesema, Claudia Coipan, John W. A. Rossen, Indra L. Bergval, Eelco Franz

**Affiliations:** 1 Centre for Infectious Disease Control (CIb), National Institute for Public Health and the Environment (RIVM), Bilthoven, the Netherlands; 2 Department of Medical Microbiology and Infection Prevention, University of Groningen, University Medical Center Groningen, Groningen, the Netherlands

**Keywords:** Comparative genomics, Stx2f, Human, Pigeon, Reservoir, STEC, tEPEC, Hybrid pathotypes, Clonal lineages, Pathogens

## Abstract

**Background:**

Wild birds, in particular pigeons are considered a natural reservoir for *stx*_2f_-carrying *E. coli*. An extensive comparison of isolates from pigeons and humans from the same region is lacking, which hampers justifiable conclusions on the epidemiology of these pathogens.

Over two hundred human and pigeon *stx*_2f_-carrying *E. coli* isolates predominantly from the Netherlands were analysed by whole genome sequencing and comparative genomic analysis including in silico MLST, serotyping, virulence genes typing and whole genome MLST (wgMLST).

**Results:**

Serotypes and sequence types of *stx*_2f_-carrying *E. coli* showed a strong non-random distribution among the human and pigeon isolates with O63:H6/ST583, O113:H6/ST121 and O125:H6/ST583 overrepresented among the human isolates and not found among pigeons. Pigeon isolates were characterized by an overrepresentation of O4:H2/ST20 and O45:H2/ST20.

Nearly all isolates harboured the locus of enterocyte effacement (LEE) but different *eae* and *tir* subtypes were non-randomly distributed among human and pigeon isolates.

Phylogenetic core genome comparison demonstrated that the pigeon isolates and clinical isolates largely occurred in separated clusters. In addition, serotypes/STs exclusively found among humans generally were characterized by high level of clonality, smaller genome sizes and lack of several non-LEE-encoded virulence genes. A bundle-forming pilus operon, including *bfpA*, indicative for typical enteropathogenic *E. coli* (tEPEC) was demonstrated in 72.0% of the *stx*_2f_-carrying serotypes but with distinct operon types between the main pigeon and human isolate clusters.

**Conclusions:**

Comparative genomics revealed that isolates from mild human disease are dominated by serotypes not encountered in the pigeon reservoir. It is therefore unlikely that zoonotic transmission from this reservoir plays an important role in the contribution to the majority of human disease associated with *stx*_2f_-producing *E. coli* in the Netherlands. Unexpectedly, this study identified the common occurrence of STEC_2f_/tEPEC hybrid pathotype in various serotypes and STs. Further research should focus on the possible role of human-to-human transmission of Stx2f-producing *E. coli*.

**Electronic supplementary material:**

The online version of this article (10.1186/s12864-019-5635-z) contains supplementary material, which is available to authorized users.

## Background

Shiga toxin-producing *Escherichia coli* (STEC) is a group of bacterial pathogens whose infection in humans is associated with varying clinical manifestations, including diarrhoea, haemorrhagic colitis and (occasionally fatal) haemolytic uremic syndrome (HUS) [1]. The production of Shiga toxin (Stx1 and/or Stx2 variants) is a cardinal virulence factor of this group of pathogens [2]. STEC is generally considered zoonotic with ruminants, and in particular cattle and sheep, as the main reservoirs [3, 4]. In addition, there is evidence for birds, dogs, horses and pigs being additional reservoirs and/or spill-over hosts for STEC [5]. This implies that there may be other epidemiologically relevant sources of human STEC infection beyond ruminants.

STEC harbouring the *stx*_2f_ variant are frequently found in pigeons [6–9] and occasionally in other bird species [10], but have never been reported in ruminants. Initially, *stx*_2f_-carrying *E. coli* were thought to be pigeon adapted with a limited impact on disease in humans. However, reports from several countries imply that infections with *stx*_2f_-carrying *E. coli* are more common than anticipated [11–13]. In the Netherlands, they constituted 16% of all STEC infections in the period 2008–2011 but infections were generally associated with a relative mild course of the disease [13, 14].

The occurrence of *stx*_2f_-carrying strains in pigeons as well as in humans is suggestive for these birds being a zoonotic reservoir for human infection. Whole genome characterisation and strain comparison indicated that *stx*_2f_-carrying *E. coli* from pigeons, humans with mild disease, and HUS patients belonged to three distinct sub-populations [15] with a certain but limited overlap between pigeon and human isolates with respect to serotypes and MLST [9, 11]. Whether this overlap is sufficient to explain the epidemiological situation and justify the conclusion that human clinical isolates originate from a pigeon reservoir (directly by strains or indirectly by phages) remains under debate. To date, an extensive comparison of isolates from pigeons and humans from the same region is lacking, which hampers justifiable conclusions on the epidemiology of *stx*_2f-_carrying *E. coli*. With this study, an in-depth genomic comparison of *stx*_2f-_carrying *E. coli* from pigeons and humans from the Netherlands is provided.

## Results

### In silico analyses; typing

**Fig. 1:**
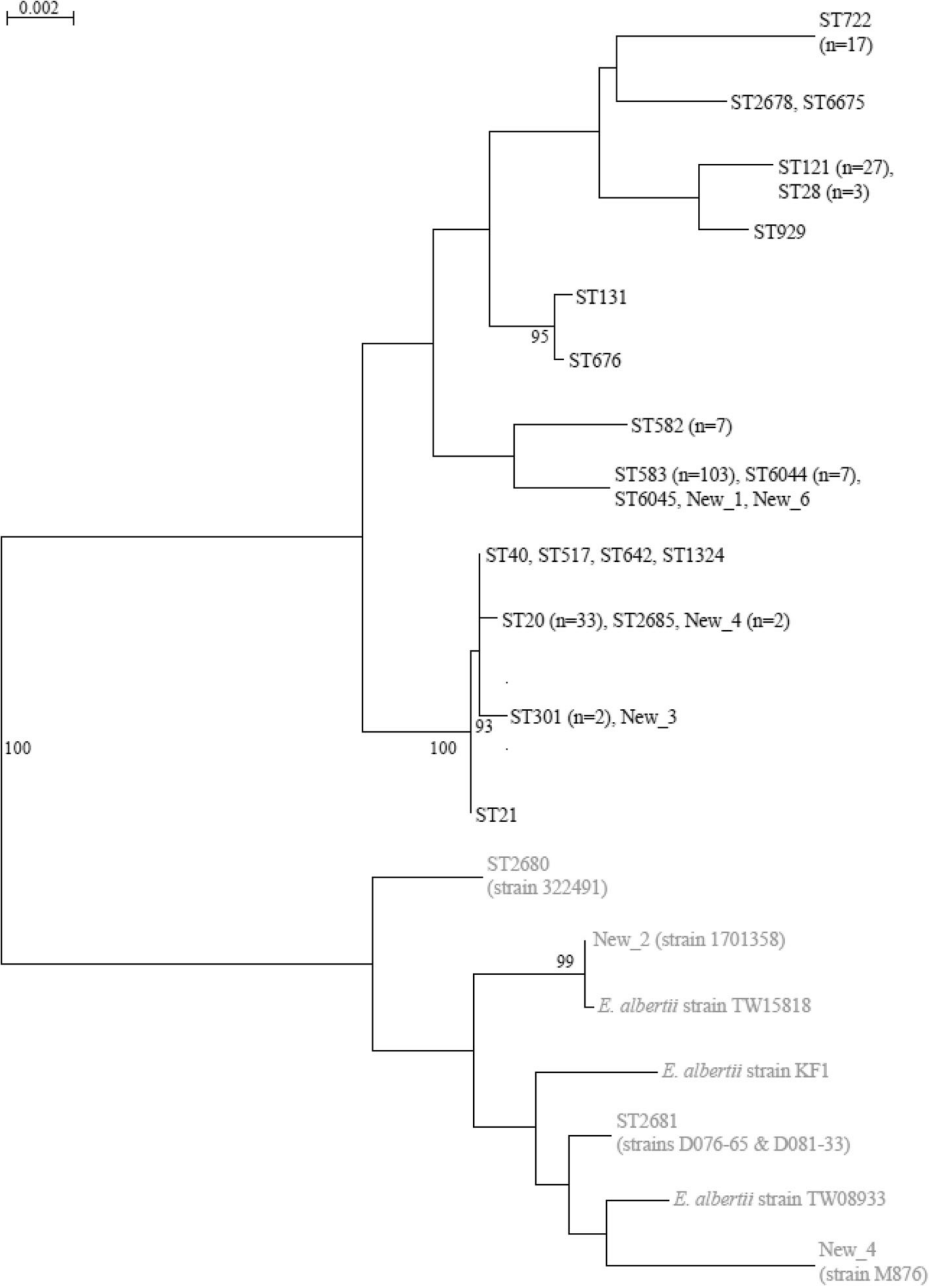
Maximum-likelihood phylogeny of *rpoB* gene sequences representing the 223 *stx*_2f_-carrying isolates and including three *E. albertii* controls. Branches representing *E. coli* are given in black, while *E. albertii* are indicated in grey. Bootstrap values of more than 90% are indicated

Analysis of the *rpoB* gene was used to confirm that the *stx*_2f_-carrying isolates were really *E. coli* and not *E. albertii* as some authors have suggested [16]. In silico *rpoB* screening and phylogenetic analysis of the resulting alignment demonstrated that nearly all *stx*_2f_-carrying isolates included in this study were *E. coli* except for four Dutch isolates (two human and two pigeon) and one from the UK. Three of these Dutch isolates displayed an ONT:H- serotype, while the fourth was typed to O115:H52. MLST typing according to the *E. coli* scheme resulted in two known STs (ST2681 (*n* = 2) and ST2680) and two new ones (see Additional file 1), however the *rpoB* sequence of all four isolates clearly cluster them among *E. albertii* (Fig. 1)*.* A closer look at the UK strain SRR6144114 in the ENA database confirmed that this is indeed an *E. albertii* isolate rather than an *E. coli*.

The remaining 218 (*rpoB* confirmed) *E. coli* constituted of 26 different serotypes according to in silico serotyping (Table 1, Additional file 1). This concerned 21 different O-types and 13 H-types, but also several isolates that were not typeable, i.e. seven ONT:H6, two ONT:H2, one ONT:H32 and one O4:H−. The serotypes showed a strong non-random distribution among the human and pigeon sources. Serotypes O63:H6 (36.7%), O125:H6 (12.8%), O113:H6 (11.5%) and O145:H34 (8.7%) were the most prominent serotypes among human isolates and were not found among pigeon isolates. Other common serotypes, showing a limited degree of overlap between sources, included O45:H2 (6.9%), O128:H2 (6.0%) and O132:H34 (3.2%). In silico MLST revealed that sequence type (ST) 583 (47.2% (103/218)) was the most prevalent among especially human isolates of this study. ST20 was the second most often found (15.1% (33/218)), especially among the pigeon isolates. Other frequently encountered STs were ST121 (12.4% (27/218)) and ST722 (8.7% (19/218)) (Table 1, Additional file 1).

**Table 1:** In silico serotyping and MLST results of the isolates included

Country	ST Complex	ST	Serotype	Source
*Netherlands* (145)	ST-122 (69)	583 (69)	O63:H6 (42)	Human (41), Leafy greens (1)
			O125:H6 (22)	Human (22)
			ONT:H6 (5)	Human (5)
	ST-20 (15)	20 (15)	O45:H2 (7)	Pigeon (6), Human (1)
			O128:H2 (5)	Human (4), Livestock (1)
			O4:H2 (1)	Pigeon (1)
			O4:H− (1)	Pigeon (1)
			ONT:H2 (1)	Pigeon (1)
	ST-28 (30)	121 (27)	O113:H6 (25)	Human (25)
			ONT:H6 (2)	Human (2)
		28 (3)	O96:H7 (2)	Human (2)
			O81:H6 (1)	Human (1)
	ST-582 (7)	582 (7)	O132:H34 (7)	Human (6), Pigeon (1)
	None (7)	6044 (7)	O63:H6 (7)	Human (7)
	None (5)	722 (5)	O145:H34 (5)	Human (5)
	None (2)	**2681 (2)**	**ONT:H− (2)**	**Pigeon (2)**
	ST131 (1)	131 (1)	O16:H5 (1)	Human (1)
	None (9)	517 (1)	O35:H19 (1)	Human (1)
		676 (1)	O16:H5 (1)	Human (1)
		929 (1)	O166:H14 (1)	Human (1)
		1324 (1)	ONT:H32 (1)	Leafy greens (1)
		6045 (1)	O63:H6 (1)	Human (1)
		New_1 (1)	O63:H6 (1)	Human (1)
		**New_2 (1)**	**O115:H52 (1)**	**Human (1)**
		New_3 (1)	O184:H30 (1)	Pigeon (1)
		**New_5 (1)**	**ONT:H− (1)**	**Human (1)**
*Others* (78)	ST-122 (34)	583 (34)	O63:H6 (28)	Human (16), Unknown (12)
			O125:H6 (6)	Human (6)
	ST-20 (21)	20 (18)	O45:H2 (8)	Pigeon (3), Human (2), Avian (1), Pet (1), Unknown (1)
			O128:H2 (8)	Animal (1), Human (1), Pigeon (1), Unknown (5)
			O4:H2 (1)	Human (1)
			O75:H2 (1)	Pigeon (1)
		New_4 (2)	O4:H2 (2)	Pigeon (2)
		2685 (1)	ONT:H2 (1)	Pigeon (1)
	None (14)	722 (14)	O145:H34 (14)	Human (3), Animal (1), Unknown (10)
	ST-165 (2)	301 (2)	O55:H9 (1)	Human (1)
			O80:H2 (1)	Human (1)
	ST-29 (1)	21 (1)	O26:H11 (1)	Human (1)
	None (6)	40 (1)	O109:H21 (1)	Human (1)
		642 (1)	O34:H4 (1)	Human (1)
		2678 (1)	O137:H6 (1)	Human (1)
		**2680 (1)**	**O69:H52 (1)**	**Human (1)**
		6675 (1)	O137:H6 (1)	Human (1)
		New_6 (1)	O63:H6 (1)	Human (1)

Note: The number of isolates are indicated in between parentheses. The isolates indicated in bold and underlined are *E. albertii*

### In silico analyses; virulence genes

Additional to *stx*_2f_ numerous other virulence genes were identified in the *E. coli* isolates (Table 2, Additional file 2). Nearly all isolates (99.1% (216/218)) harboured the LEE island that included the following virulence genes; *eae*, *espA*, *espB*, *espF* and *tir* (Table 2, Additional file 2). Several *eae* and *tir* subtypes were detected with a non-random distribution among serotypes (Table 2). The increased serum survival gene *iss*, two colicin encoding genes (*cba* and *cma*)*,* and the non-LEE encoded type III effector *nleA* were not found at all among the serotypes only encountered among human isolates. In contrast, the non-LEE-encoded effector gene *espJ* was identified in the types exclusively found among human isolates. All 25 O113:H6 isolates showed the presence of a high pathogenicity island (HPI) which included *fyuA* (ferric yersiniabactin uptake) and five *irp* (iron-repressible protein) genes (Table 2). This HPI was also present in 12 other isolates representing eight different serotypes including O96:H7 (*n* = 2) and O137:H6 (n = 2) (Additional file 2). Two of the three HUS isolates (EF453, EF467 and EF476) also contained this PAI, although in one isolate only partially; *irp1* and *irp3* were absent (i.e. EF467 (O26:H11)).

**Table 2:** Prevalence of *E. coli* virulence genes among the eight most prevalent serotypes of the *stx*_2f_-carrying *E. coli* isolates

Gene	O63:H6	O113:H6	O125:H6	O132:H34	O145:H34	O4:H2	O45:H2	O128:H2
*astA*	77/80	24/25	24/28	4/7	12/19	4/4	15/15	13/13
*bfpA*	77/80		24/28	4/7	12/19	4/4	13/15	13/13
*cba*						4/4	13/15	10/13
*cdt-I*	80/80	25/25	28/28	7/7	19/19	4/4	15/15	9/13
*celB*	2/80							
*cif*	80/80	25/25	28/28			3/4	15/15	13/13
*cma*						4/4	14/15	10/13
*eae*	80/80	25/25	28/28	7/7	19/19	4/4	14/15	12/13
-subtype	alpha2	beta2	alpha2	alpha2	iota1	beta1	beta1&iota1	beta1
*ehly1*				6/7				
*espA*	80/80	25/25	28/28	7/7	19/19	4/4	14/15	12/13
*espB*	80/80	25/25	28/28	7/7	19/19	4/4	14/15	12/13
*espC*	77/80		28/28	7/7	18/19			
*espF*	80/80		27/28	7/7	19/19	4/4	14/15	12/13
*espJ*	80/80	25/25	28/28					
*fyuA*		25/25						
*irp1*		25/25						
*irp2*		25/25						
*irp3*		25/25						
*irp4*		25/25						
*iss*	1/80					4/4	15/15	13/13
*nleA*						3/4	15/15	13/13
*nleB*	2/80		27/28	7/7	19/19	4/4	14/15	13/13
*nleC*	72/80			1/7		4/4	15/15	13/13
*stx*_2f_	80/80	25/25	28/28	7/7	19/19	4/4	15/15	13/13
*tccP*	4/80			2/7				
*tir*	76/80	25/25	28/28	4/7	19/19	4/4	14/15	12/13
-subtype	gamma1	alpha1	gamma1	gamma1	alpha1	beta1	beta1	beta1
*vat*		24/25						

Two different allelic variants of the enteroaggregative *Escherichia coli* heat-stable enterotoxin (EAST1) gene *astA* were found. Most *astA* positive *E. coli* isolates (84.1% (159/189)) had the allelic variant that was described before (accession number: AB042002 (Additional file 2)) and were in a few instances linked to incF plasmid genes (see BFP section). However, all the O113:H6 (*n* = 25), one O109:H21 and six other HPI positive isolates contained an AB042002 variant with a non-synonymous mutation (G67A) resulting in an amino acid change (A23T) in the AstA protein (Additional file 2). In 72.0% of the O113:H6 isolates *astA* was located on a large contig (average size 105,170 nt) that also contained numerous incI1 conjugative transfer protein genes like *traA*-*C*, *traE*-*F*, *traH-I*, *traN*-*Q*, *traU*, *traW*-*Y*.

**Fig. 2:**
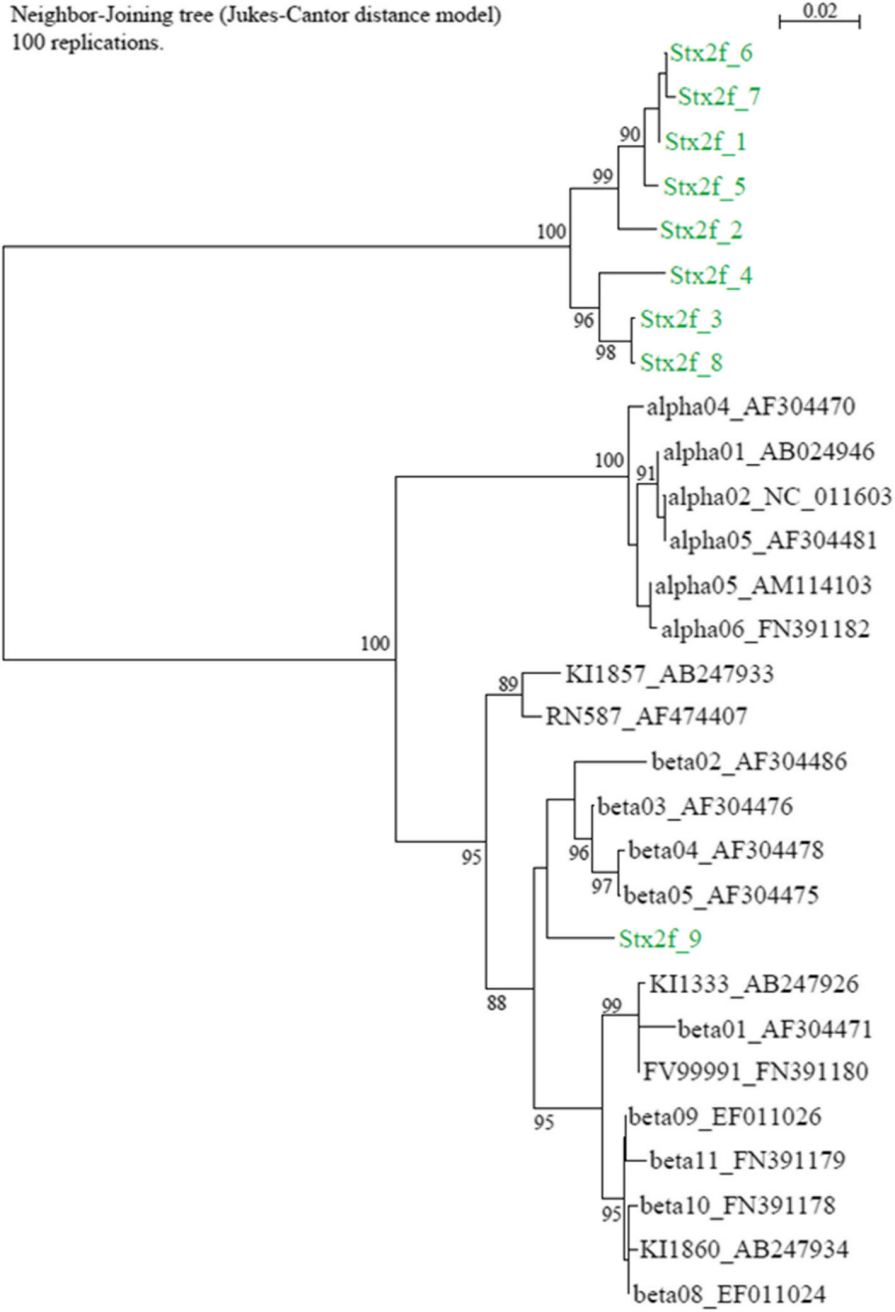
Maximum-likelihood phylogeny of known *bfpA* alleles, together with ones encountered in this study

Surprisingly, the major structural subunit of bundle-forming pilus determinant *bfpA* was demonstrated in the majority of pigeon and human isolates (Table 2). In total, 72.0% (157/218) of these STECs harboured this typical enteropathogenic *E. coli* (tEPEC) determinant. However, none of the O113:H6 isolates (n = 25) nor the three Italian HUS isolates contained *bfpA* (Table 2, Additional file 2). In total nine different *bfpA* alleles were identified in the entire isolate set investigated. Eight of them belonged to a different subgroup clearly separated from the well-known alpha and beta subtypes (Fig. 2, Additional files 2 and 3). In addition, one novel beta allele was characterized in two strains.

### Comparative genomics and phylogenetic analysis

**Fig. 3:**
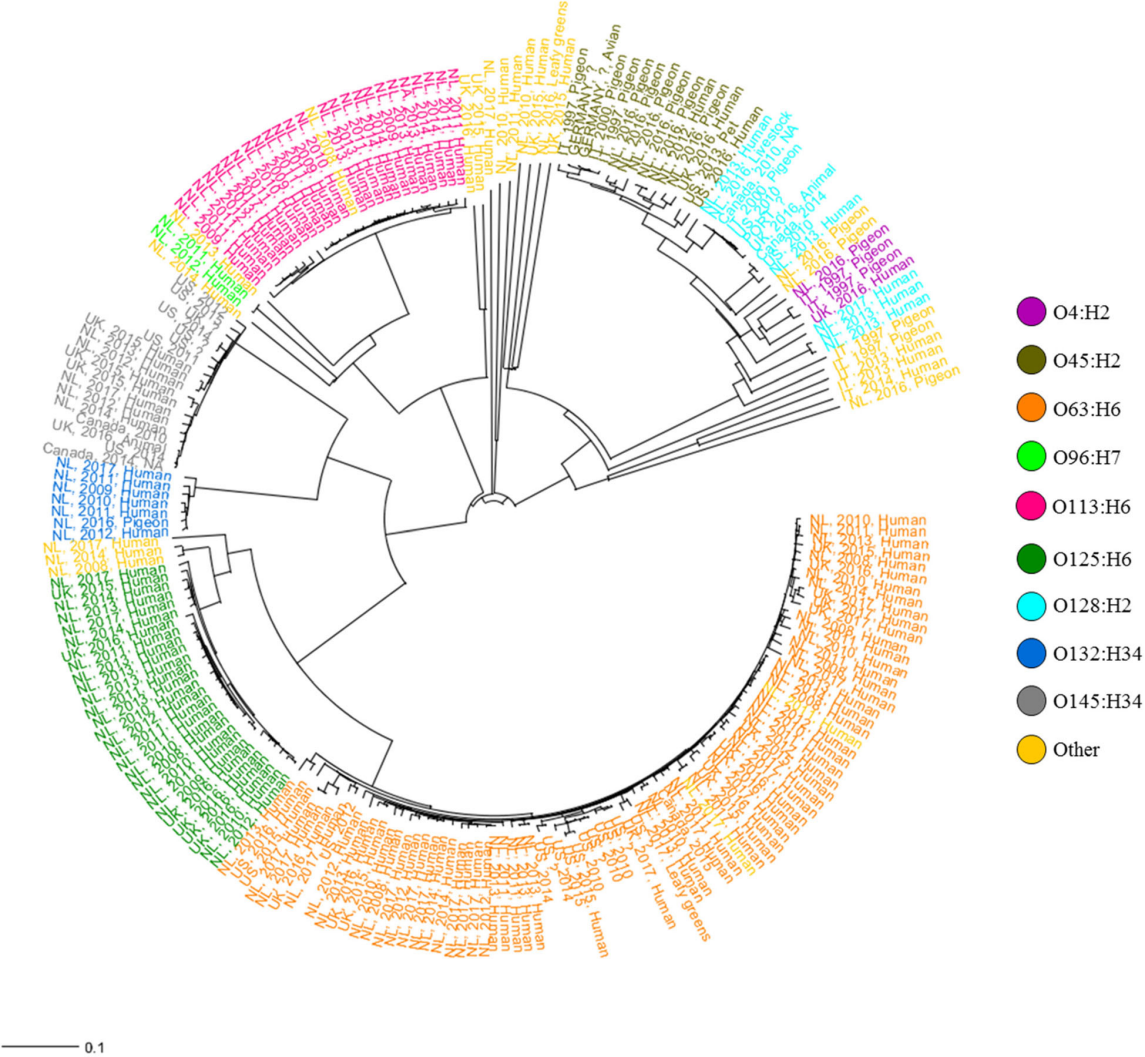
Neighbor-Joining phylogenetic tree of 218 *stx*_2f_-carrying *E. coli* isolates based on wgMSLT data. The phylogenetic tree is constructed on a distance matrix calculated from the different allele numbers of the wgMLST scheme. The colours represent the various serotypes. Each isolate is indicated by the country of isolation, the year of isolation and its origin

Comparative whole genome MLST (wgMLST) analysis of the 218 *stx*_2f_ -carrying *E. coli* predominantly displayed a clustering of the isolates according to serotype/ST with a general clustering of pigeon and human isolates along the phylogenetic tree which follows the observation of a clear non-random distribution of serotypes/STs among human and pigeon isolates (Fig. 3). The phylogenetic tree also showed shorter branch lengths of the serotypes exclusively found in humans (O63:H6, O113:H6, O125:H6) compared to the others that show overlap in occurrence between humans and pigeons (O4:H2, O45:H2 and O128:H2), indicative for a stronger clonal relation among the types exclusively found in humans. This was confirmed by looking in more detail at the number of genes different within the top eight serotypes investigated in this study (Table 3). The strict human associated types showed significant lesser number of different genes (T-test, *P* = 0.022) and smaller average distance between isolates in comparison to the other types. (T-test, *P* = 0.011). The pathogenicity island LEE was shown to be present in nearly all *E. coli* isolates included in the study (*n* = 212). It encoded the intimin adhesin gene *eae*, but also the well-known effector proteins EspB, EspF, EspG, EspH, EspZ, Map and Tir. Phylogenetic analysis zooming in on the 42 genes of LEE only, displayed a very similar clustering as wgMLST analysis (Additional file 4: Figure S1).

**Table 3:** Overview of the gene differences among the top eight serotypes investigated

Serotype	Strains (n)	wgMLST genes shared	Total number of different genes^1^	Average number of different genes per isolate	Average distance within a serotype
O63:H6	80	4092	1333	16.7	78.8
O113:H6	25	4126	358	14.3	62.1
O125:H6	28	4026	308	11.0	33.4
O132:H34	7	4031	167	23.9	52.9
O145:H34	19	4016	502	26.4	78.0
O4:H2	4	3793	545	136.3	303.7
O45:H2	15	3793	519	34.6	117.2
O128:H2	13	3851	1097	84.4	361.0

Note: ^1^ Identical genes among a serotype were excluded

Over 60 genes belonged to the *stx*_2f_-phage including important determinants like *cro*, *cI*, int, capsid and tail structural genes and packaging genes. However, some of the genes normally involved in infection and propagation of Stx phages, such as *cII*, *cIII*, N Q, O, and P seemed to be absent. Consequently, immunological VERO cells test assays were performed to determine whether the phage was active. Shiga toxin-production was confirmed for a selection (*n* = 18) of strains belonging to various serotypes (data not shown). The phylogeny of over 60 *stx*_2f_-phage associated genes revealed a more scattered distribution of the various serotypes (Additional file 4: Figure S2).

**Fig. 4:**
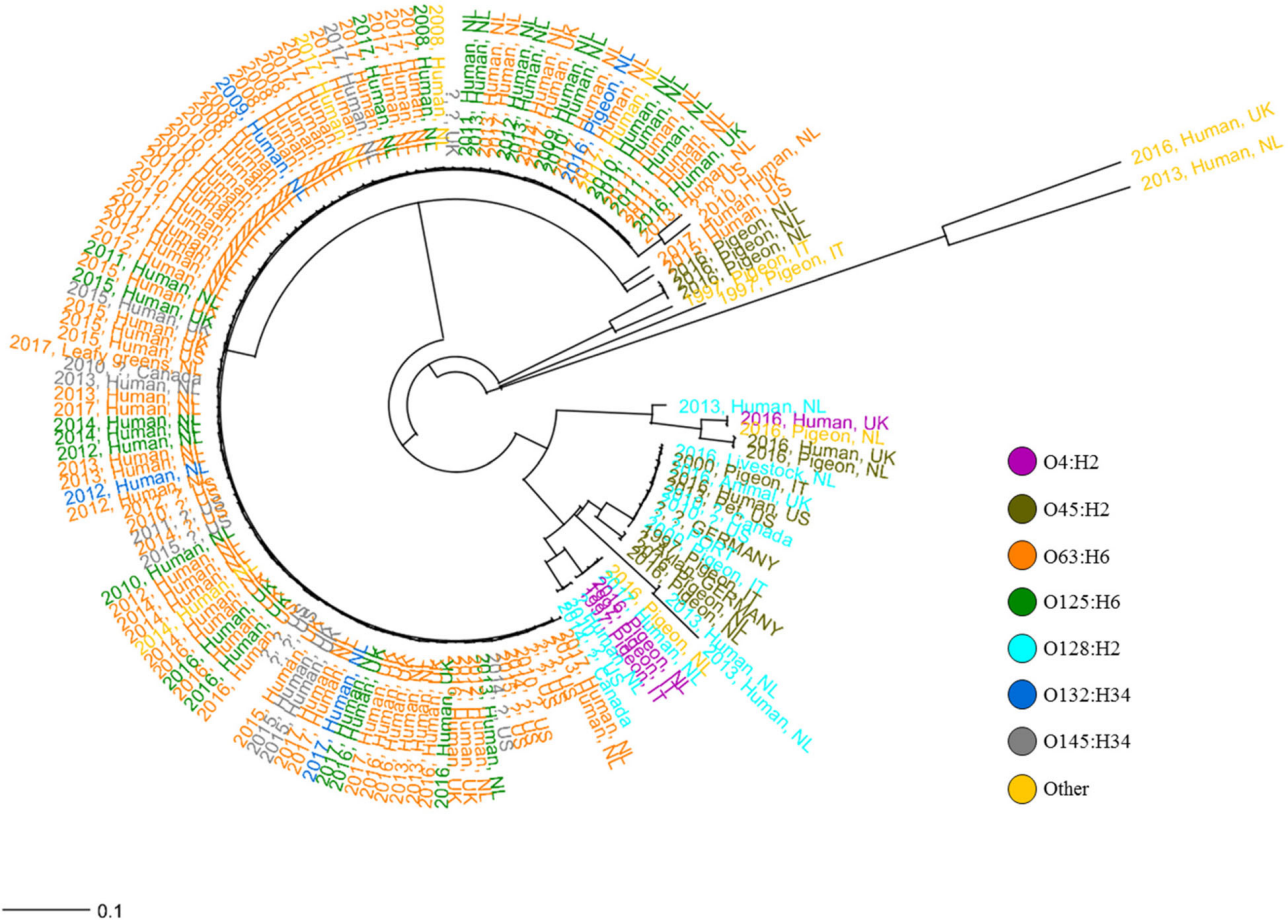
Neighbor-Joining phylogenetic tree of 157 *stx*_2f_-carrying *E. coli* isolates harbouring a BFP plasmid. The tree is based on the 14 genes of the BFP operon and the colours represent the various serotypes. Each isolate is indicated by the country of isolation, the year of isolation and its origin

The unexpected result of the high prevalence of the *bfpA* gene among *stx*_2f_-carrying *E. coli* isolates required subsequent genetic studies (RAST and BLAST). This revealed that *bfpA* was located in a cluster of 14 genes, i.e. the bundle-forming pilus (BFP) operon. Phylogenetic analysis of this operon showed a clear separate clustering of O4:H2, O45:H2, and O128:H2 from the rest of the serotypes (Fig. 4).

The global regulator elements of BFP the so-called *perABC* (also known as *bfpTVW*) was not found in any of the *bfpA* positive strains.

Since BFP is commonly associated with FIB/FIIA plasmid families, this association was also investigated*.* In silico analysis revealed that in all BFP positive isolates the FIB *repA* gene was present and was almost always located on the same contig as *bfpA,* suggesting these genes were co-localized on the same plasmid. Because the assemblies concern draft genomes the FIIA *repA* was not always on the same contigs as FIB *repA* and *bfpA*. Consequently, it is not known whether these FIIA *rep* genes are part of the BFP plasmid. In addition to *repA*, various other specific incF plasmid genes were encountered like the conjugative transfer protein genes *traB*-*D*, *traF*-*I*, *traN*, *traP*-*R*, *traU*-*X*, *trbA*-*F* and *trbI*.

### In silico analyses; genome size

**Fig. 5:**
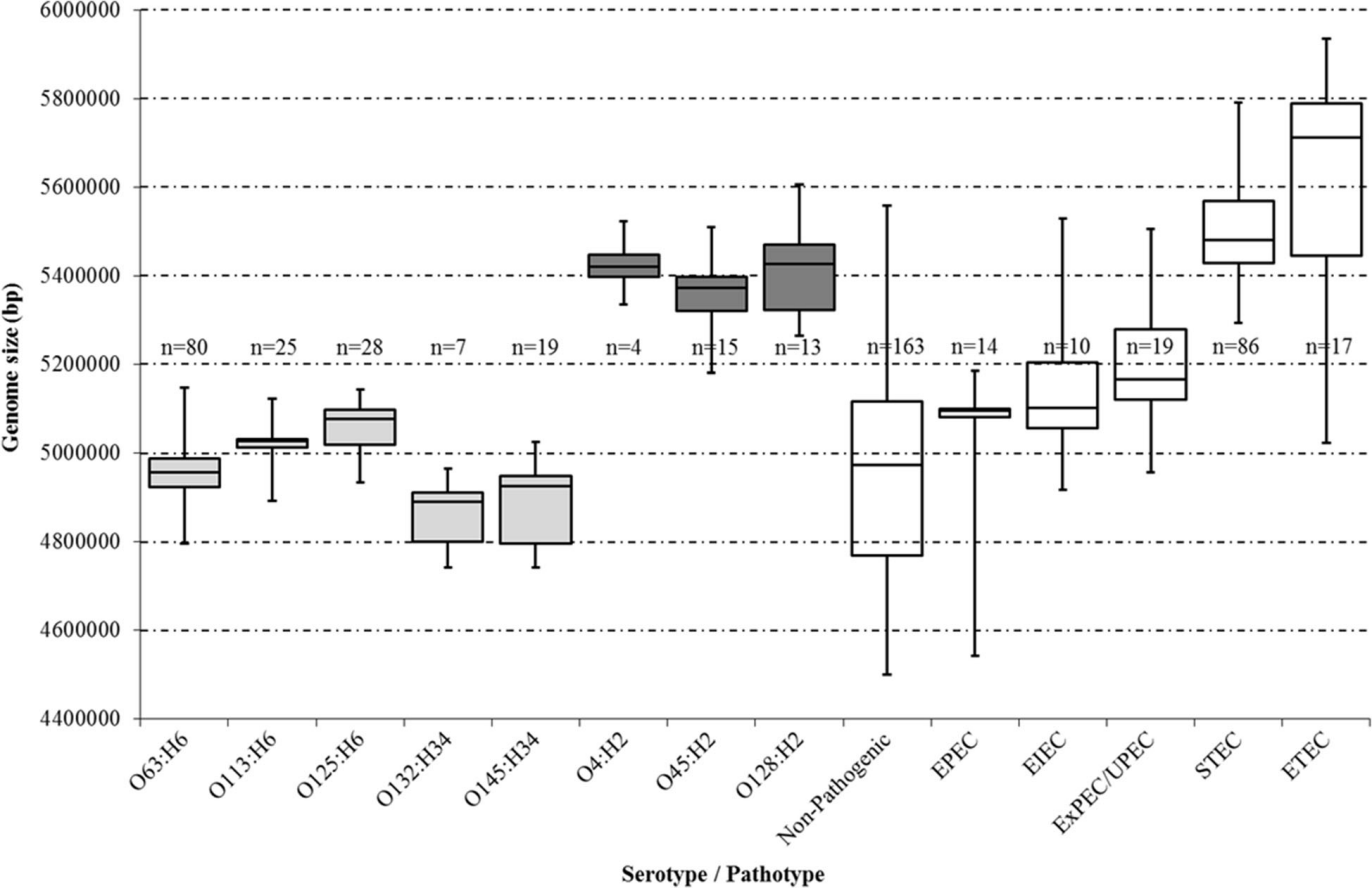
Genome sizes of the most prevalent *stx*_2f_-carrying *E. coli* serotypes in comparison to various publicly available *E. coli* pathotypes (enterobase.warwick.ac.uk/species/index/ecoli). The numbers within the figure show the isolates included in each group. The light grey boxplots represent the human associated serotypes, while the dark grey ones show predominantly pigeon isolates. The white boxplots display various *E. coli* pathotypes; aEPEC: atypical Enteropathogenic *E. coli,* EIEC: Enteroinvasive *E. coli,* ExPEC; Extraintestinal pathogenic *E. coli*, UPEC: Uropathogenic *E. coli*, STEC: Shiga-toxin producing *E. coli*, ETEC: Enterotoxigenic *E. coli*

A marked difference in genome sizes of the isolates was identified. The genomes of the serotypes O63:H6, O125:H6, O113:H6, O132:H34 and O145:H34 were similar in size to non-pathogenic *E. coli* and enteropathogenic *E. coli* (EPEC). In contrast, the serotypes O4:H2, O45:H2 and O128:H2 were more comparable to genome sizes of STECs and enterotoxigenic *E. coli* (ETEC) (Fig. 5, Additional file 1).

## Discussion

Earlier studies emphasized the existence of a strict association between STEC carrying the *stx*_2f_ gene and pigeons, with limited impact on disease on humans [6, 7, 17]. However, reports from several countries imply that infections with *stx*_2f_-carrying *E. coli* are more common than anticipated [11–13]. In the Netherlands, *stx*_2f_-carrying *E. coli* constituted 16% of all STEC infections in the period 2008–2011, but were generally associated with a relative mild course of the disease [13]. As several STEC assays targeting *stx* genes are not capable of detecting the 2f variant, limited data on *stx*_2f_-carrying *E. coli* from human infections in other countries are available due to under-diagnosis [18]. The aim of the present study was to investigate to which extent *stx*_2f_-carring *E. coli* from pigeons and humans are genetically related and consequently whether pigeons could be considered a plausible source of transmission to humans. Based on comparative genomics this study provides several lines of evidence for the existence of generally separate *stx*_2f_-carrying *E. coli* populations in humans and pigeons. First, there is very limited overlap in serotypes among human and pigeon isolates. The isolates from humans are dominated by serotypes that are not encountered among pigeons. Second, the strict human associated types and the other types (found predominantly in pigeons and sporadically in humans) largely form two distinct phylogenetic clusters based on wgMLST, LEE island, and the BFP operon. Third, the strict associated human types, in contrast to the other types, tend to be highly clonal. Fourth, the genomic characteristics of the strict human associated types and pigeon types differ regarding genome size and virulence factor composition. In addition, an unexpected but important finding of the present study was that the majority of the *stx*_2f_-carrying *E. coli* (72.0%) carried cardinal genes for tEPEC (BFP operon) as well as for STEC (*stx*_2f_), suggesting the existence of hybrid STEC/tEPEC strains.

STEC serotypes can be strongly associated with specific reservoirs [4]. Besides a report on the isolation from shellfish and the associated production water (possibly contaminated with urban wastewater) [19] the dominant *stx*_2f_-carying serotype O63:H6 in the present study has regularly and exclusively been reported from humans [11, 13, 14, 20]. A weakness of the presented data is the possible under-sampling of the pigeon reservoir, which could have resulted in an underestimation of the circulating diversity. This was statistically confirmed by rarefaction analysis (Additional file 5). However, a probability analysis showed that if the common Dutch strict human associated serotypes (O63:H6, O113:H6, O125:H6, O145:H34) do actually occur in the pigeon reservoir with the same distribution as among humans we would have isolated them even with the current sample size (Additional file 5). The absence of these serotypes in pigeons and wild birds confirms the finding of a few other studies, although it was not clear whether this always concerned STECs [9, 21, 22]. Together with the observed high level of clonality, this strongly suggests that these common human associated *stx*_2f_-carrying strains are not originating from the pigeon reservoir.

In this study, the majority of the STEC strains (carrying *stx*_2f_) is identified to simultaneously be tEPEC (defined by the presence of *bfpA*). The presence of both *bfpA* and *stx*_2f_ in *E. coli* strains is not new since it has been reported before. For example, Hazen et al. [23] demonstrated both genes in a human O128:H2 strain (STEC_H.1.8), which has been included in the current study. In addition, very recently, a study was published by Gioia-Di Chiacchio et al. [24], describing O137:H6 strains from a cockatiel and a budgerigar carrying both *bfpA* and *stx*_2f_. However, our present study describes the occurrence of STEC/tEPEC hybrids on a far larger scale and among various *E. coli* serotypes and in different phylogenetic groups. The serotypes O63:H6, O125:H6, O132:H34 and O145:H34 all have been described earlier as (typical) EPEC [11, 25, 26]. While atypical EPEC (i.e. LEE-positive, *bfpA*-negative, *stx*-negative) have both animal and human reservoirs, tEPEC have a strict human reservoir [27, 28]. In addition, tEPEC is most often not associated with typical severe STEC symptoms like bloody diarrhea and HUS but seems to be linked to milder but more persistent symptoms [13, 27, 29], which is similar as observed for *stx*_2f_-carrying *E. coli* infections [13, 27, 29]. Surprisingly, the results of the present study demonstrated that also the majority of pigeon associated strains were identified as STEC/tEPEC hybrids. However, as described in this study the genomes of pigeon and human hybrid STEC/tEPEC show considerable differences. First, the genome sizes of the hybrids belonging to the strict human associated serotypes were generally smaller and more resembling EPECs while the hybrids belonging to other serotypes were significantly larger and more resembling STECs. Second, similar to Grande et al. [15], several non-LEE encoded type III effector STEC virulence determinants (*nleA, nleB* and *nleC*) were demonstrated only in strains from the pigeon associated cluster (including a limited number of human isolates) and in the clinical HUS isolates, while absent from the majority of the human associated hybrids associated with relatively mild disease. Although strains commonly encountered in relatively mild disease among humans are not found in the pigeon reservoir, some overlap between pigeons and humans can be seen regarding the more typical virulent STEC strains. Finally, pigeon and human isolates showed clear distinct BFP operon types.

Altogether, the emerging picture suggests that the *stx*_2f_-carrying *E. coli* and *stx*_2f_/tEPEC hybrids commonly encountered in relatively mild human disease do not directly originate from the pigeon reservoir. Although sporadically isolated from other sources it is possible that these mild disease strains do not have a zoonotic reservoir at all in terms of an animal species in which the pathogen is maintained and shed. Similarly no animal reservoirs have been identified for other STEC hybrids like *stx*-EAEC O104:H4 [30–32] and *stx*-ExPEC O80:H2 [33, 34], which also show strong clonal relations [35]. In addition, it was demonstrated that the strain involved in an outbreak of STEC O117:H7 linked to transmission among men who have sex with men was characterized by a significantly smaller genome size compared to STEC O157 and O26 [36]. Moreover, the genomic relationships were consistent with existing symptomatic evidence for chronic infection with this O117:H7 serotype.

## Conclusions

Pigeons should not be regarded as the most likely direct source of the most frequent encountered *stx*_2f-_carrying *E. coli* types encountered in relatively mild human disease. Humans themselves may be the more plausible reservoir for the majority of milder infections with this pathogen. This study also showed the unexpected common existence of STEC/tEPEC hybrids among pigeon and human isolates although in different reservoir dependent genomic backbones (i.e. genome size, virulence genes, BFP operon type). The occurrence of the BFP plasmid among non-human isolates should be further investigated with respect to whole plasmid sequence and patho-phenotype of the BFP-carrying pigeon isolates. Possibly a phylodynamic approach would be helpful in elucidating the spread and evolution of this plasmid between isolates of different host species. Phylodynamic studies may also be of value in studying the possible human-to-human transmission of Stx2f-tEPEC hybrids. Finally, further experimental research on the infectivity of the Stx2f phages to *E. coli* isolates of different sources and of different pathotypes may be informative on their potential spread.

## Methods

### Stx2f –carrying *E. coli* strains

Most of the Dutch human isolates (*n* = 119) originated from the collection held at the National Institute for Public Health and the Environment in the Netherlands (RIVM) and were collected as part of the national surveillance programme (2008–2017) [13]. Some additional isolates originated from the STEC-ID-net study and were isolated from the faeces of hospitalized patients or patients visiting their GP with (bloody) diarrhoea (*n* = 10) [14]. Thirteen Dutch pigeon isolates included in this study were obtained from a small study among pigeon droppings in the Netherlands in 2016. In total 140 pigeon faeces were sampled for the presence of Stx2f-producing *E. coli* according to ISO/TS 13136:2012. A prevalence of 9.3% was found among racing pigeons as well as free living pigeons in urban environments (data not shown). Two leafy green and one livestock isolates were also included in the study.

Besides Dutch isolates international ones were also included in this study in order to provide genomic context (*n* = 78). Raw reads or assemblies of non-Dutch isolates were recovered from publicly available databases; European Nucleotide Archive (ENA (www.ebi.ac.uk/ena)) and *Escherichia*/*Shigella* Enterobase (enterobase.warwick.ac.uk/species/ecoli/). The Italian isolates (n = 11) originated from a previous study [15]. An overview of the 223 isolates included in this study and their characteristics can be found in Additional file 1.

### Whole genome sequencing

The sequencing of the Dutch strains was performed on various Illumina platforms (Illumina, San Diego, CA, USA), i.e. MiSeq PE300, HiSeq 2000 and HiSeq 2500 with the appropriate Illumina library protocols.

Raw reads were trimmed and de novo assembled using CLC Genomics Workbench v 10.0 (Qiagen, Hilden, Germany). The parameters for trimming were as follows: ambiguous limit, 3; quality limit, 0.05; number of 5 = −terminal nucleotides, 1; number of 3 = −terminal nucleotides, 1. The parameters for the de novo assembly were as follows: mapping mode, create simple contig sequences (slow); bubble size, 50; word size, 20; minimum contig length, 200 bp; perform scaffolding, yes; auto-detect paired distances, yes.

### Assembly statistics and genome size analysis

The assemblies were assessed using the assembly file statistics of SeqSphere^+^ 4.1.9 software (Ridom GmbH, Münster, Germany [37]). Various characteristics were determined like contig count, N50 and genome sizes.

To compare genome sizes of the various *stx*_2f_-carrying *E. coli* serotypes against those of different *E. coli* pathotypes the *Escherichia*/*Shigella* Enterobase database was consulted. The following pathotypes aEPEC (atypical enteropathogenic *E. coli*), EIEC (enteroinvasive *E. coli*), ETEC (*enterotoxigenic E. coli*), ExPEC (extraintestinal pathogenic *E. coli*), STEC (Shiga-toxin producing *E. coli*) and UPEC (uropathogenic *E. coli*) were looked up as searches in the Field “Simple Patho” via the enterobase.warwick.ac.uk/species/ecoli/search_strains (this search was performed on 01-03-2018). Genome sizes of the selected pathotypes were registered and together with the *stx*_2f_-carrying *E. coli* serotypes were compared by box plot analysis.

### In silico MLST analysis, serotyping and determination of virulence and antimicrobial resistance genes

Individual gene phylogeny of *rpoB* was generated after in silico analysis of this determinant and extraction of the nucleotide sequences using SeqSphere^+^. An alignment and maximum-likelihood tree using the Kimura [38] two-parameter model of distance estimation was made using Seaview Version 4.5.4 [39].

In silico multilocus sequence typing (MLST) analysis was performed on the seven well-known housekeeping genes for *E. coli*, i.e. *adk*, *fumC*, *gyrB*, *icd*, *mdh*, *purA* and *recA* [40]. Allelic variants of these seven gene loci were identified using SeqSphere^+^. Allele numbers and sequence types (STs) were assigned according to the *E. coli* MLST database (mlst.warwick.ac.uk/mlst/dbs/Ecoli).

In silico serotypes were determined using the SeqSphere+ software by screening the assemblies for the presence of O-type (*wzm*, *wzt*, *wzx* and *wzy*) and H-type genes (*fliC*) as previously described [41].

Additionally the assemblies were analysed for the presence/absence of *E. coli* virulence genes. The sequence information for most of these genes was retrieved from the Center for Genomic Epidemiology database (bitbucket.org/account/user/genomicepidemiology/projects/DB), but some gene clusters were added from own local databases and literature searches, e.g. *bfpA*, *cdtI*-*cdtV*, *espB*. Again SeqSphere+ was used to screen the assemblies for over a hundred virulence genes (see [42]).

### Comparative genomics and phylogenetic analysis

KmerFinder 2.4 [43] (cge.cbs.dtu.dk/services/KmerFinder/) was used to determine the best matching *E. coli* isolates to the seven most prevalent serotypes of the *stx*_2f_-carrying isolates. The best matches were *E. coli* O18:H7 strain IHE3034 (NC_017628.1, 5,108,383 bases) with 5179 genes with coding sequences (CDS) and *E. coli* O103:H2 strain 12009 (NC_013353.1, 5,449,314 bases), 5698 genes with CDS. Both complete genomes (strain IHE3034 as reference and strain 12009 as query) were used to design a whole genome multilocus sequence typing (wgMLST) scheme with the SeqSphere+ software to determine the genomic relatedness of the *E. coli* isolates included in this study. The target scan procedure details were set to 90% required identity and 100% required percentage aligned to the reference sequence. In total, 3365 targets were defined for core genome MLST (3,221,601 bases), while 1401 were assigned as accessory targets (1,111,383 bases). Overall, 413 targets were discarded because either homologous genes were encountered or they were missing a stop codon.

The assemblies of at least one representative of each serotype included in this study was annotated by RAST [44]. These RAST annotations were used to investigate certain areas of the genome, like the pathogenicity island locus of enterocyte effacement (LEE), *stx*_2f_–phage and a bundle-forming pilus (BFP) plasmid, in more detail. The annotations helped to determine the composition of these genetic elements and enabled phylogenetic analysis after extraction of these specific parts from the assemblies. First, the contigs where the genes of interest were located, were recovered from the assemblies of the *stx*_2f_-carrying isolates. For the LEE island this concerned the intimin determinant *eae*, for the *stx*_2f_-phage the two *stx* subunits and in the case of the BFP plasmid the major bundle-forming pilus gene *bfpA*. Next the length of these contigs was determined. The longest contigs in the most common serotypes of the *stx*_2f_-carrying isolates were used to assess the genomic structures of each of these three areas. Basic local alignment search tool (BLASTn) analyses were also included in this analysis [45]. In this way, the 42 genes which compose the LEE island of the various *stx*_2f_-carrying *E. coli* serotypes were identified and used to develop a MLST scheme with SeqSphere+. Over 60 genes were characterized to belong to the *stx*_2f_–phage. They were used to setup a core phage MLST scheme using SeqSphere+. The genes belonging to BFP plasmids were also recovered from the annotations and assemblies, resulting in a core plasmid MLST scheme of over 70 genes.

### Statistical analysis

Rarefaction analysis; To test whether the pigeon associated *E. coli* population has been sampled enough as to capture the majority of the serotypes the distinct serotypes identified among both human and pigeon isolates were counted. A rarefaction analysis, implemented in EstimateS 9 [46] was run for individual-based abundance data, with 100 runs, randomization of individuals without replacement, and extrapolation to 500 individuals. The result is expressed as curves of the estimated number of serotypes expected to be found for a particular sample size, with associated confidence intervals (Additional file 4: Figure S2).

Bayesian inference of expected proportions of serotypes in pigeons; The extent to which certain serotypes could be absent in the sample retrieved from pigeons due to undersampling can be quantitatively evaluated by calculating the probability of observing more isolates of that particular serotype (p_higher in Additional file 5: Table S3) than actually observed in the sample. In the hypothesis that there is no difference between the distribution of the serotypes between humans and pigeons, the distribution of the various serotypes in the human sample was used as a beta prior to inform the binomial distribution of the isolates of the corresponding serotypes in the pigeon sample. For this, the beta binomial cumulative distribution was evaluated using the function pbetabinom.ab (q, size, shape1, shape2, log.p = FALSE) implemented in the R package VGAM [47]. Function arguments were: the number of pigeon isolates of a particular serotype (q) and the total number of isolates sampled from pigeons (size), the number of human isolates of the same particular serotype (shape1) and the number of human isolates of other serotypes (shape2). Hence, the prevalence of isolates from pigeon in a particular serotype was evaluated against the prevalence of the same serotype in the human sample.

## Additional files

Additional file 1: **Table S1.** Characteristics of all 223 *stx*_2f_-carrying strains analysed in this study; accession number, country, source, isolation year, in silico serotype, in silico ST, *rpoB* sequence analysis and assembly statistics. (XLSX 43 kb)

Additional file 2: **Table S2.** Absence/Presence of *E. coli* virulence factors in 223 *stx*_2f_-carrying strains. (XLSX 119 kb)

Additional file 3: Fasta format of the *bfpA* gene sequences detected in this study. (FASTA 5 kb)

Additional file 4: **Figure S1.** Neighbor-Joining phylogenetic tree of 212 LEE-positive *E. coli* isolates based on the 42 genes of locus of enterocyte effacement. The colours represent the various serotypes. Each isolate is indicated by the country of isolation, the year of isolation and its origin. **Figure S2.** Neighbor-Joining phylogenetic tree of 218 stx2f-carrying *E. coli* isolates based on the core phage MLST data. The colours represent the various serotypes. Each isolate is indicated by the country of isolation, the year of isolation and its origin. (DOCX 1158 kb)

Additional file 5: **Figure S3.** Rarefaction analysis. The two lines indicate the number of serotypes (S (est) on the y-axis) relative to the sample size (number of samples on the x-axis), with the observed values represented by the continuous lines and the extrapolated values represented by the dotted lines. The confidence intervals are marked by the shaded areas. **Table S3.** Bayesian inference. The Table summarizes the observed number of isolates of each particular serotype in both the human and pigeon samples. The probability of observing a higher number of isolates in the pigeon sample, given that the serotypes distribution was the same as for the human samples, is given in the *p_higher* column for each of the respective serotypes. (DOCX 60 kb)

## Abbreviations

BFP: Bundle-Forming Pilus
BLAST: Basic Local Alignment Search Tool
EIEC: Enteroinvasive *E. coli*
ENA: European Nucleotide Archive
EPEC: Enteropathogenic *E. coli*
ETEC: Enterotoxigenic *E. coli*
ExPEC: Extraintestinal pathogenic *E. coli*
HPI: High Pathogenicity Island
HUS: Haemolytic Uremic Syndrome
LEE: Locus of Enterocyte Effacement
RAST: Rapid Annotation using Subsystem Technology
ST: Sequence Type
STEC: Shiga toxin-producing *E. coli*
tEPEC: typical Enteropathogenic *E. coli*
UPEC: Uropathogenic *E. coli*
wgMLST: whole genome MLST
