# Supplementary figures and images for: Comparative genomics reveals a lack of evidence for pigeons as a main source of stx2f-carrying Escherichia coli causing disease in humans and the common existence of hybrid Shiga toxin-producing and enteropathogenic E. coli pathotypes
Source: BMC Genomics. 2019 Apr 5;20:271. doi: 10.1186/s12864-019-5635-z (PMC6451237; doi:10.1186/s12864-019-5635-z)

**Figure S1**


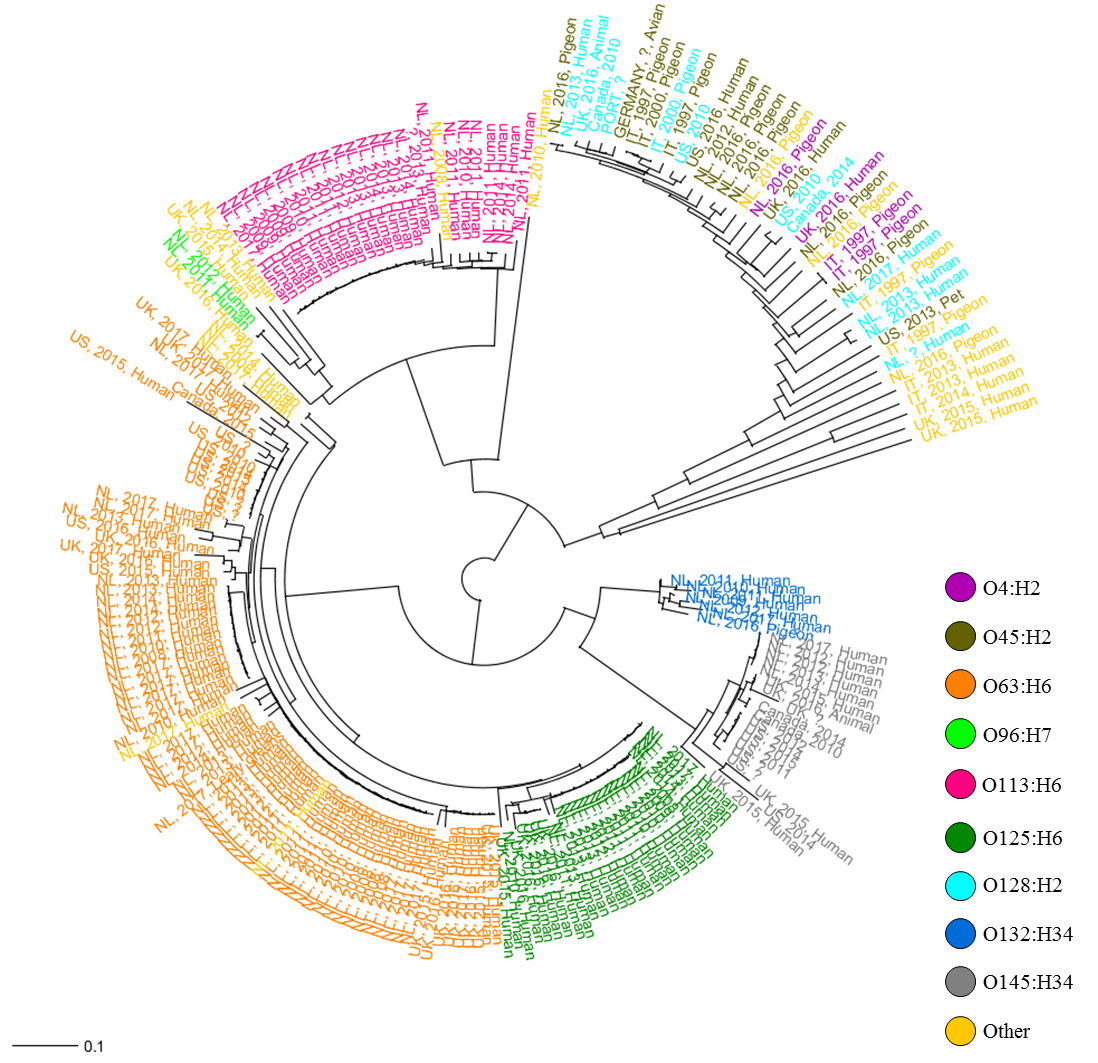


**Figure S2**


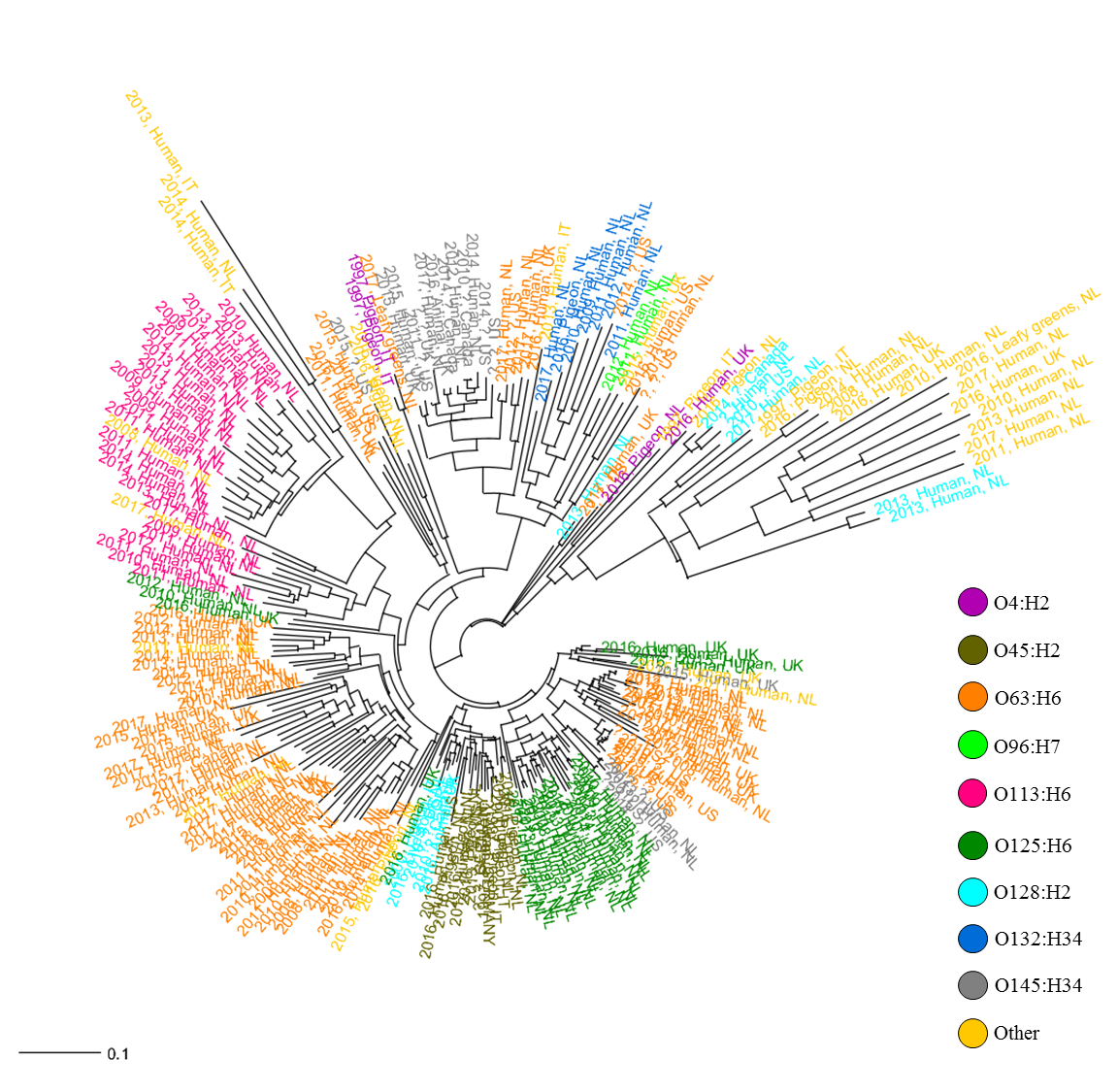

Supplement: Supplementary file 4 — Figure S1. Neighbor-Joining phylogenetic tree of 212 LEE-positive E. coli isolates based on the 42 genes of locus of enterocyte effacement. The colours represent the various serotypes. Each isolate is indicated by the country of isolation, the year of isolation and its origin. Figure S2. Neighbor-Joining phylogenetic tree of 218 stx2f-carrying E. coli isolates based on the core phage MLST data. The colours represent the various serotypes. Each isolate is indicated by the country of isolation, the year of isolation and its origin. (DOCX 1158 kb) [file 12864_2019_5635_MOESM4_ESM.docx]
