# Supplementary material for: Comparative genomics reveals a lack of evidence for pigeons as a main source of stx2f-carrying Escherichia coli causing disease in humans and the common existence of hybrid Shiga toxin-producing and enteropathogenic E. coli pathotypes
Source: BMC Genomics. 2019 Apr 5;20:271. doi: 10.1186/s12864-019-5635-z (PMC6451237; doi:10.1186/s12864-019-5635-z)
Supplement: Supplementary file 5 — Figure S3. Rarefaction analysis. The two lines indicate the number of serotypes (S (est) on the y-axis) relative to the sample size (number of samples on the x-axis), with the observed values represented by the continuous lines and the extrapolated values represented by the dotted lines. The confidence intervals are marked by the shaded areas. Table S3. Bayesian inference. The Table summarizes the observed number of isolates of each particular serotype in both the human and pigeon samples. The probability of observing a higher number of isolates in the pigeon sample, given that the serotypes distribution was the same as for the human samples, is given in the p_higher column for each of the respective serotypes. (DOCX 60 kb) [file 12864_2019_5635_MOESM5_ESM.docx]

**Figure S3**


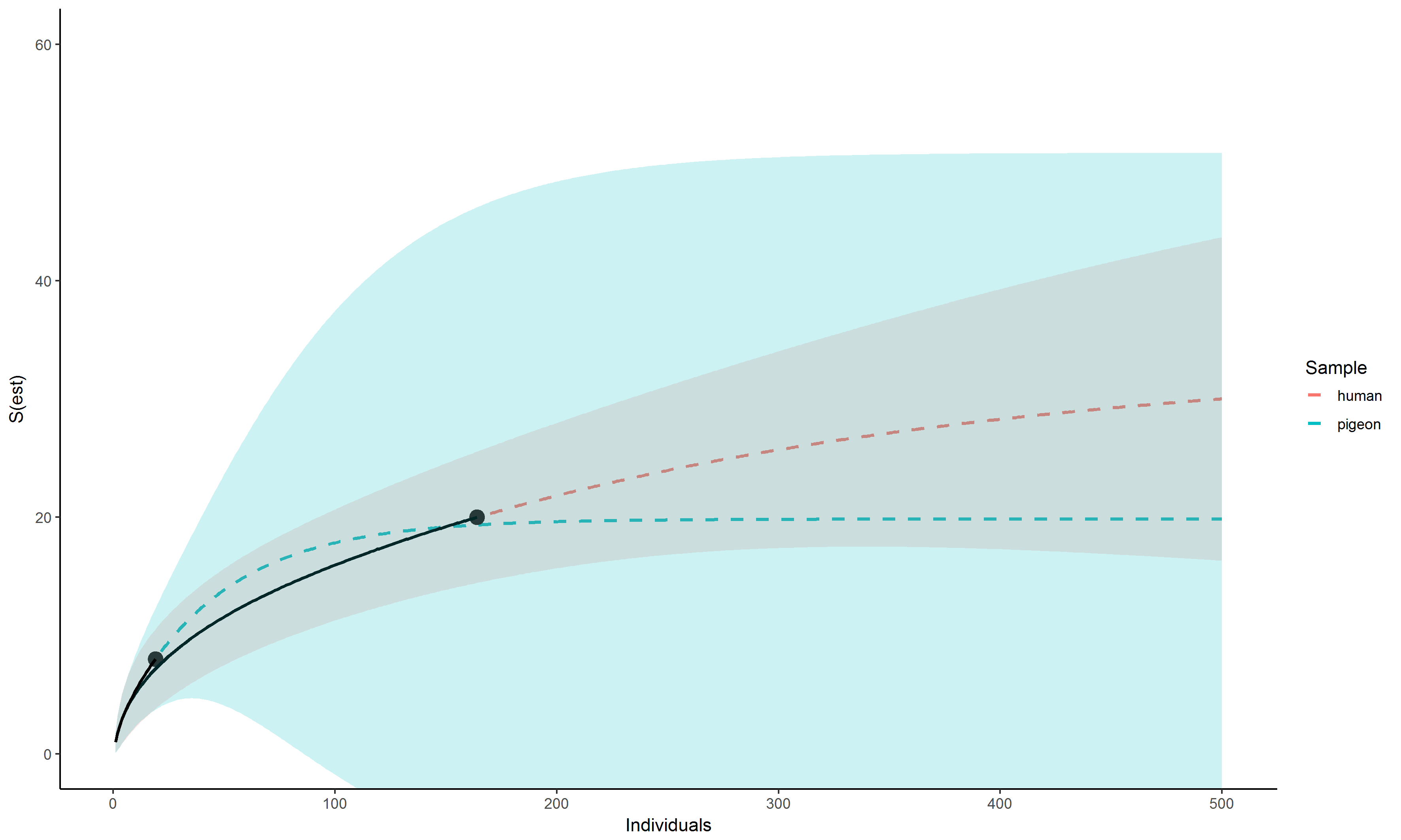


**Table S3**

| Serotype | Human | Pigeon | *p_higher* |
| --- | --- | --- | --- |
| O63:H6 | 67 | 0 | 0.999 |
| O125:H6 | 28 | 0 | 0.965 |
| O113:H6 | 25 | 0 | 0.948 |
| O145:H34 | 8 | 0 | 0.593 |
| ONT:H6 | 7 | 0 | 0.544 |
| O132:H34 | 6 | 1 | 0.158 |
| O128:H2 | 5 | 1 | 0.120 |
| O45:H2 | 3 | 9 | <0.001 |
| O16:H5 | 2 | 0 | 0.198 |
| O96:H7 | 2 | 0 | 0.198 |
| O137:H6 | 2 | 0 | 0.198 |
| O4:H2 | 1 | 3 | <0.001 |
| O26:H11 | 1 | 0 | 0.104 |
| O34:H4 | 1 | 0 | 0.104 |
| O35:H19 | 1 | 0 | 0.104 |
| O55:H9 | 1 | 0 | 0.104 |
| O80:H2 | 1 | 0 | 0.104 |
| O81:H6 | 1 | 0 | 0.104 |
| O109:H21 | 1 | 0 | 0.104 |
| O166:H14 | 1 | 0 | 0.104 |
| O4:H− | 0 | 1 | NA |
| O75:H2 | 0 | 1 | NA |
| O184:H30 | 0 | 1 | NA |
| ONT:H2 | 0 | 2 | NA |
